# Role of Autophagy in the Microenvironment of Oral Squamous Cell Carcinoma

**DOI:** 10.3389/fonc.2020.602661

**Published:** 2020-12-09

**Authors:** Daniel Peña-Oyarzún, Montserrat Reyes, María Paz Hernández-Cáceres, Catalina Kretschmar, Eugenia Morselli, Cesar A. Ramirez-Sarmiento, Sergio Lavandero, Vicente A. Torres, Alfredo Criollo

**Affiliations:** ^1^ Advanced Center for Chronic Disease (ACCDiS), Facultad de Ciencias Químicas & Farmacéuticas and Facultad de Medicina, Universidad de Chile, Santiago, Chile; ^2^ Facultad de Odontología, Instituto de Investigación en Ciencias Odontológicas, Universidad de Chile, Santiago, Chile; ^3^ Autophagy Research Center, Universidad de Chile, Santiago, Chile; ^4^ Departamento de Fisiología, Facultad de Ciencias Biológicas, Pontificia Universidad Católica de Chile, Santiago, Chile; ^5^ Departamento de Patología y Medicina Oral, Facultad de Odontología, Universidad de Chile, Santiago, Chile; ^6^ Facultades de Ingenieria, Medicina y Ciencias Biológicas, Institute for Biological and Medical Engineering, Pontificia Universidad Católica de Chile, Santiago, Chile; ^7^ Cardiology Division, Department of Internal Medicine, University of Texas Southwestern Medical Center, Dallas, TX, United States

**Keywords:** oral squamous cell carcinoma, autophagy, tumor microenvironment, cancer, carcinoma-associated fibroblast

## Abstract

Oral squamous cell carcinoma, the most common type of oral cancer, affects more than 275,000 people per year worldwide. Oral squamous cell carcinoma is very aggressive, as most patients die after 3 to 5 years post-diagnosis. The initiation and progression of oral squamous cell carcinoma are multifactorial: smoking, alcohol consumption, and human papilloma virus infection are among the causes that promote its development. Although oral squamous cell carcinoma involves abnormal growth and migration of oral epithelial cells, other cell types such as fibroblasts and immune cells form the carcinoma niche. An underlying inflammatory state within the oral tissue promotes differential stress-related responses that favor oral squamous cell carcinoma. Autophagy is an intracellular degradation process that allows cancer cells to survive under stress conditions. Autophagy degrades cellular components by sequestering them in vesicles called autophagosomes, which ultimately fuse with lysosomes. Although several autophagy markers have been associated with oral squamous cell carcinoma, it remains unclear whether up- or down-regulation of autophagy favors its progression. Autophagy levels during oral squamous cell carcinoma are both timing- and cell-specific. Here we discuss how autophagy is required to establish a new cellular microenvironment in oral squamous cell carcinoma and how autophagy drives the phenotypic change of oral squamous cell carcinoma cells by promoting crosstalk between carcinoma cells, fibroblasts, and immune cells.

## Introduction

The study of the tumor microenvironment has gained attention during the last decade and the development of effective anti-cancer therapies has been challenging (1). Tumors are not just masses of growing cells, but a novel tissue with evolving features over time (2). Tumors contain, besides primary tumor cells, stromal cells (including fibroblasts, vascular endothelial cells and adipocytes, among others) and immune cells (such as macrophages, T cells and B cells), which assist tumor initiation and/or progression (2).

Oral squamous cell carcinoma (OSCC) is the most common type of oral cancer and current treatments are limited to surgery, radiotherapy, chemotherapy or a combination of these, inflicting a huge impact on the quality of life of the patients, such as speech impairment, swallowing difficulties and face remodeling (3). Understanding the cellular mechanisms that allow communication between primary tumor cells and the tumor microenvironment can be crucial to find novel pharmacological targets for the treatment of OSCC. One of these mechanisms is autophagy, an intracellular degradation process that behaves as a “double-edge sword” when it comes to cancer (4). Treatment of different types of cancer, including OSCC, with autophagy modulators yields either promising or devastating results (5). Here we summarize and discuss the evidence regarding the role of autophagy in the communication of OSCC cells and their microenvironment. This review will hopefully shed some light on the contradictory results of autophagy-based treatments in OSCC.

## Oral Squamous Cell Carcinoma

Oral cancer is one of the head and neck cancers which represent the sixth most common malignancy in the world (6, 7). Oral cancer affects nearly 300,000 persons each year, mainly in high-income countries (8, 9). Oral cancer is twice as common in men as in women and the average age for diagnosis is 62, although it also occurs in younger people (10). Importantly, only 40%–50% of patients have a 5-year survival, and if there is metastasis, the average 5-year survival rate is 39%. However, survival is 84% at 5 years if the diagnosis is made at an early stage. Therefore, early diagnosis of oral cancer is a decisive factor in improving patient survival (10).

**Figure 1:**
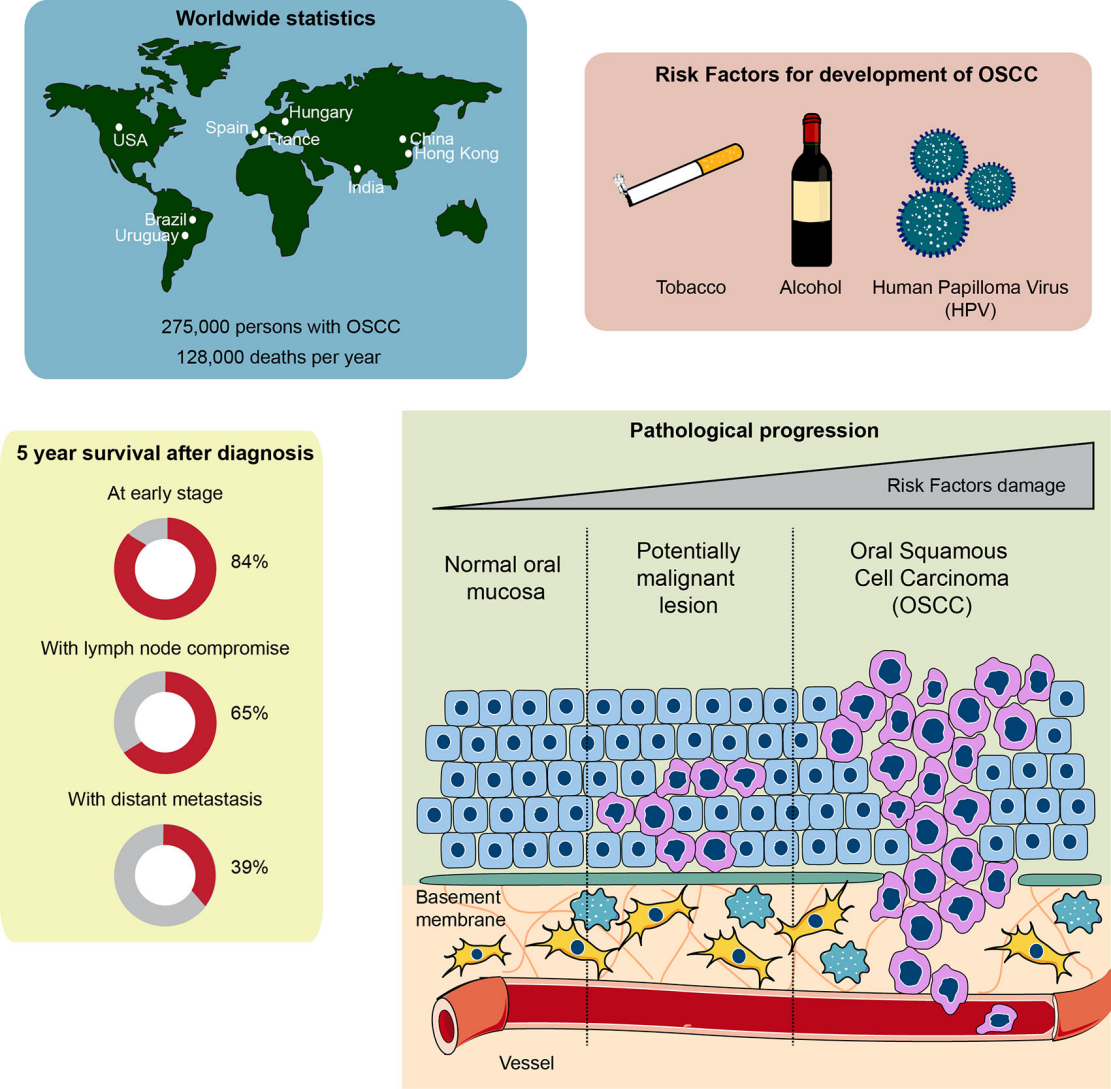
Global statistics, survival rate, and pathophysiological features of oral squamous cell carcinoma (OSCC). Top left: countries with higher cases of OSCC diagnosed around the world. Top right: the main risk factors involved in OSCC development and progression. Bottom left: chances of survival 5 years after being diagnosed with OSCC. Note that early diagnosis of OSCC is crucial to ensure over 80% survival chance after 5 years. The general statistics show a 50% survival rate after 5 years, given that OSCC is usually diagnosed late. Bottom right: development of OSCC from a normal oral epithelium. The normal epithelium, composed of epithelial cells known as keratinocytes, is located over a basement membrane that separates the epithelium from the connective tissue composed of fibroblasts, immune cells and vessels. Exposure to carcinogens derived from the risk factors of the top right panel generate a potentially malignant lesion, characterized by an altered cellular morphology that starts affecting the inner layers of the epithelium close to the basement membrane, progressing toward the outer layers of the epithelium. Continuous exposure to carcinogens leads to OSCC development, a phenomenon that alters all the epithelial cell layers both genetically and morphologically. Interplay between connective tissue cells and OSCC cells is also observed, which assists OSCC growth and metastasis.

About 90% of oral cancers originate in the stratified non-keratinized epithelium of the oral mucosa, which is the reason for its denomination as oral squamous cell carcinoma. Its main risk factors include consumption of tobacco and alcohol, along with other possible risk factors, such as chronic irritation, poor oral hygiene, human papillomavirus (HPV), malnutrition and immune system suppression (11–13). These risk factors provoke the development of various genetic instabilities and molecular alterations, including the loss of heterozygosity of chromosomes 3, 4 7, 8, 11, 17, and 19, among others, down-regulation of tumor-suppressor genes such as TP53, RB, CDKN2A, and up-regulation of oncogenes such as cyclin D1 (6, 14, 15). Following exposure to the carcinogens mentioned above, normal oral keratinocytes form an epithelial dysplasia, which is a tissue alteration where cells adapt to stressful stimuli by changing their number and shape. Continuous exposure to carcinogens shifts the progression of the epithelial dysplasia from mild to severe, ending with its malignant transformation to OSCC and metastasis (16). Metastasis of OSCC cells is mainly through the lymphatic vessels to the cervical lymph nodes on the same side of the face, which plays a critical role in the management and prognosis of patients with OSCC. The most common distant metastatic sites are the lungs, liver and bones (17, 18). The pathological progression, as well as the global burden and survival chances after diagnosis of OSCC, are depicted in **Figure 1**.

The clinical presentation of OSCC is highly variable; the most common is observed as an ulcerated lesion in the oral cavity that does not heal, containing harsh edges by palpation, while other signs may include mobile teeth, bleeding, pain or numbness in the mouth or face (9, 10). Treatment options are limited to surgical resection as a primary treatment, and radiation as a primary treatment or as an adjuvant after surgery. Chemotherapy is used mainly as adjuvant after surgery, given that pharmacological treatments usually have secondary effects (19). In most cases surgery, radiation and chemotherapy lead to negative effects on the patient’s quality of life, such as speaking impairment, swallowing dysfunction, physical appearance alteration, sensory disability and chronic pain (19, 20). Thus a deeper understanding of the cellular and molecular biology of OSCC, regarding its development and progression, is required to improve pharmacological treatments and avoid secondary effects.

In this context, it has been acknowledged that the tumor microenvironment plays a vital role in OSCC progression and invasion, as it directly affects both tumor growth and its ability to progress and metastasize. Here, blood vessels, nerves and immune cells contribute to the tumor heterogeneity limiting therapeutic access, altering drug metabolism and contributing to drug resistance (21).

## The Microenvironment of the OSCC

Stromal cancer-associated fibroblasts, CAFs, are the primary non-immune infiltrative cells in the carcinoma microenvironment (22). Studies have revealed that CAFs show increased expression of proteins involved in actin cytoskeleton remodeling during migration, such as Rho-associated coiled-coil kinase 2, ROCK2 (23), focal adhesion kinase, FAK (24), and alpha smooth muscle actin, α-SMA (25). *In vitro* studies reported that CAFs show higher migration rates compared to fibroblasts obtained from normal subjects (26), suggesting that events linked to the epithelial-mesenchymal transition (EMT) in CAFs may participate during OSCC progression. EMT of CAFs is related to increased levels of platelet-derived growth factor receptor β, PDGF-Rβ, in the plasma membrane (27), which in turn activates Janus kinase 2, JAK2, and the signal transducer and activator of transcription protein 3, STAT3 (25). Activation of the JAK2/STAT3 pathway in CAFs provokes the release of epidermal growth factor, EGF, which promotes the EMT in tumor cells (25). It has also been reported that CAFs release other factors that contribute to the EMT in tumor epithelial cells. Among these, CAFs generate exosomes containing microRNAs (miRs) such as miR-382-5p (28), which is associated with advanced TNM stages of the OSCC (29). Although the molecular mechanism by which miR-382-5p affects OSCC has not been totally elucidated, studies have shown that miR-382-5p is required to down-regulate the expression of the Myc-competitor MAD (MDX1) in breast cancer (30), as well as the expression of the negative regulator of cell motility Deleted in Liver Cancer, DLC-1, in hepatic cancer (31), suggesting that miR-382-5p may reduce the expression of tumor suppressor genes in OSCC. However, it is worth noting that OSCC-related CAFs may also reduce the delivery of specific miRs such as miR-34a-5p, which has been shown to reduce the expression of the tyrosine kinase receptor AXL, decreasing β-catenin-dependent proliferation and SNAIL-dependent expression of metalloproteinases 2 (MMP2) and 9 (MMP9) (32). This reveals that OSCC-related CAFs selectively promote the release of pro-tumoral miRs over anti-tumoral miRs.

OSCC cells promote the release of several chemokines from CAFs, leading either to immune infiltration or changes in OSCC phenotype toward a pro-migratory and proliferative phenotype. For instance, OSCC cells release interleukin-1β, IL1β, which in turn provokes the release of the chemokine (C-C motif) ligand 7, CCL7, from CAFs (33). Then CCL7 binds to the chemokine (C-C motif) receptors 1-3, CCR1-3, located in the OSCC cells, increasing cell migration *in vitro* (33). The chemokine CCL2, also known as monocyte chemoattractant protein-1, MCP-1, is released by CAFs, being positively associated with lymph node metastasis (24). CCL2 positive CAFs are observed at the lymphoid metastatic focus, specifically at the marginal sinus of OSCC (34). The activation of NFκB and STAT3, as a result of hypoxia in the tumor niche, can also induce expression and release of CCL2 from CAFs (35). On the other hand, hypoxia has been shown to promote the expression of galectin-1, a protein involved in FAK activation and migration (36). Notably, galectin-1 is required for CCL2 expression in CAFs, promoting OSCC tumor growth and intravasation in xenograft models (37).

The transformation of normal fibroblasts into CAFs is also mediated by molecules that are released from OSCC cells. For instance, IL1β expression becomes progressively increased in OSCC cells and is released, activating the NFκB pathway in fibroblasts that induces release of the chemokine (C-X-C motif) ligand 1, CXCL1 (38). CXCL1 generates an autocrine mechanism that transforms fibroblasts into high-α-SMA expressing CAFs (39), suggesting that early carcinogenesis events provoke slight inflammatory alterations in the epithelial cells that then lead to the generation of CAFs.

Infiltration of immune cells is observed in OSCC, mainly promoted by the cytokines released from CAFs. Tumor-associated neutrophils, TANs, and tumor-associated macrophages, TAMs, have been observed both at the primary tumor site and at the lymphoid metastatic focus (34). Studies have shown that anti-inflammatory mediators such as TGFβ and IL10 are released from CAFs, which prevent proliferation of T-cells and promote infiltration of CD163-positive TAMs (40, 41). The CD163 membrane marker of M2 macrophages is expressed during resolution of inflammation, indicating that infiltration of specialized immune cell triggers an immunosuppressive environment (42). Thus M2 macrophage infiltration may promote tumor angiogenesis and metastasis through the release of vascular endothelial growth factor, VEGF, and PDGF (43). The presence of CD163 TAMs correlates with lymph node invasion and poor prognosis of patients with OSCC (40, 44). TAMs may also induce OSCC cells proliferation *via* the release of EGF (45). Interestingly, both CAFs and OSCC cells depict reduced TGFβ receptors 2 and 3, suggesting that the cytostatic effect of TGFβ only affects immune cells (46, 47). Finally, CAFs also attract regulatory T-cells, T-regs, shutting down the inflammatory response of T-cells and sustaining the immunosuppressive environment of the tumor (48). As in the recruitment of TAMs, the attraction of T-regs to the OSCC primary site is mediated by TGFβ and IL10 (49).

In contrast to the immunosuppressive role of CAFs, most molecules released from OSCC cells, such as IL8 and IL6, depict pro-inflammatory effects as a result of the stressful insults (i.e. tobacco smoking) (50–52). Similar to the anti-inflammatory molecules released from CAFs, IL8 and IL6 are produced in an NFκB-dependent manner, suggesting that inhibition of the NFκB pathway may be suitable for the treatment of OSCC (50, 52). The contrasting inflammatory behavior of OSCC cells and CAFs may provide an alternative approach for the treatment of OSCC, since CAF-independent growth of OSCC cells may not be sustainable in time because it would alert the defensive branch of the immune system. Further studies are required to elucidate this.

**Figure 2:**
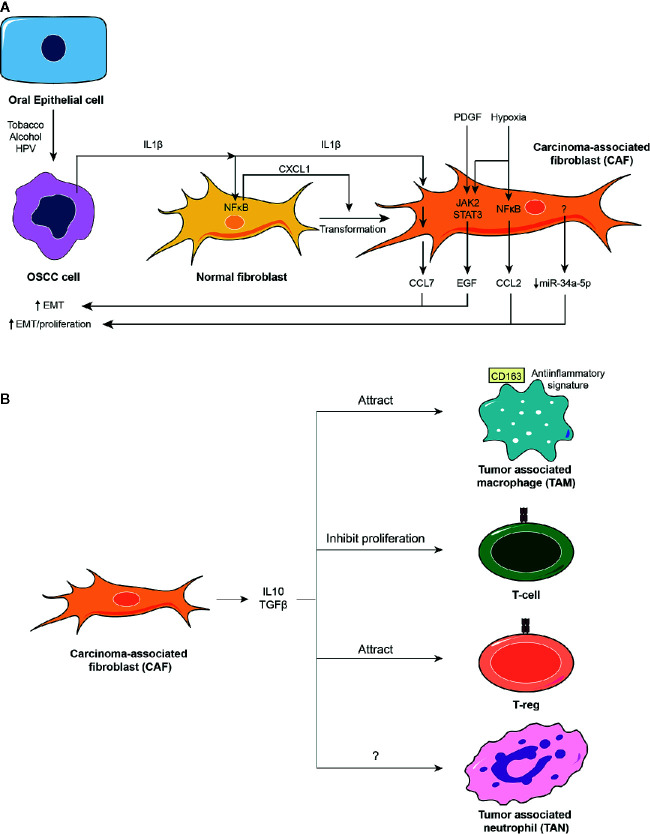
The oral squamous cell carcinoma (OSCC) tumor microenvironment. **(A)** The OSCC tumor microenvironment is mainly composed of cancer-associated fibroblasts (CAFs). CAFs derive from normal fibroblasts after autocrine stimulation of chemokine (C-X-C motif) ligand (CXCL1) chemokine through a nuclear factor κB (NFκB)-dependent mechanism. NFκB is activated in fibroblasts by the action of interleukin-1β (IL1β) released from OSCC cells. Conditions surrounding the tumoral tissue, such as increased levels of platelet-derived growth factor (PDGF) and IL1β or hypoxia activate Janus kinase (JAK)/STAT and NFκB pathways in CAFs, which induce the release of chemokines (CCL2 and CCL7) and epithelial growth factor (EGF), or inhibit the release of selective miRs such as miR-34a-5p. All these mediators augment proliferation and EMT of OSCC cells. **(B)** CAFs release well known anti-inflammatory molecules such as IL10 and TGFβ that attract anti-inflammatory macrophages and regulatory T-cells, T-regs, and inhibit proliferation of T-cells.

The OSCC microenvironment is subjected to both pro- and anti-inflammatory mediators over time. Initial epithelial insults result in increased tumor features with underlying inflammation. Pro-inflammatory cytokines released by OSCC cells provoke the development of CAFs from normal fibroblasts, thereby sustaining proliferation, migration and invasion of the OSCC cells. Also, CAFs cause the infiltration of immune cells with immunosuppressive behavior, further assisting during the metastatic process of OSCC toward lymph nodes. These antecedents are summarized in **Figure 2**.

## Autophagy in Stress and Cancer

Autophagy is a cellular process, conserved from yeasts to mammals, that promotes the degradation of wasted intracellular materials such as macromolecules and organelles, to maintain the cell homeostasis (53). A basal autophagic tone is present in all cells, but autophagy is up-regulated under certain stress stimuli to cope with the damage (54). For instance, oral gingival cells exposed to tobacco smoke, alcohol consumption or HPV increase autophagy as a protective mechanism (55). Autophagy occurs with the formation of double-membrane vesicles known as autophagosomes. Autophagosomes sequester the material that is targeted for degradation, and ultimately fuse with the lysosome to form the autolysosome (56). The lysosome contains hydrolytic enzymes and a low pH that allows degradation of the materials sequestered by the autophagosome (57).

The proteins that participate during autophagy are known as autophagy-related proteins or ATGs (56). ATG8, known as Microtubule Associated Protein 1 Light Chain 3, MAP1LC3 (or just LC3), is critical for the autophagy mechanism (58). When autophagy is induced, LC3 is cleaved by the protease ATG4, forming LC3-I, and then conjugated by the ATG5-ATG7 complex with the lipid phosphatidylethanolamine, forming LC3-II (59). Then LC3-II binds to the autophagosome membrane and promotes its elongation (58). Detection of LC3 positive vesicles and LC3-II levels are usually performed to evaluate autophagy (60). Of note, autophagy is not the mere formation of autophagosomes, but also degradation of target materials within lysosomes. This is known as “autophagy flux”, which indicates the progression from sequestration to degradation of the cargo (61). Chemical compounds such as bafilomycin-A1 and chloroquine, which inhibit the fusion between autophagosomes and lysosomes, blunt autophagy, as observed by the accumulation of autophagosomes (60). This is why conclusions from LC3 data alone should be managed with caution, as it will be discussed in the following section. Other markers besides LC3 are commonly determined to help to draw appropriate conclusions, such as Sequestrosome 1, SQSTM1 (also known as p62), which is a protein that binds poly-ubiquitinated proteins and LC3, carrying the proteins into the autophagosomes (62). SQSTM1/p62 is degraded along with the targets, leading to a reduction in its level (63). In contrast, autophagy flux blockage leads to increased levels of SQSTM1/p62.

**Figure 3:**
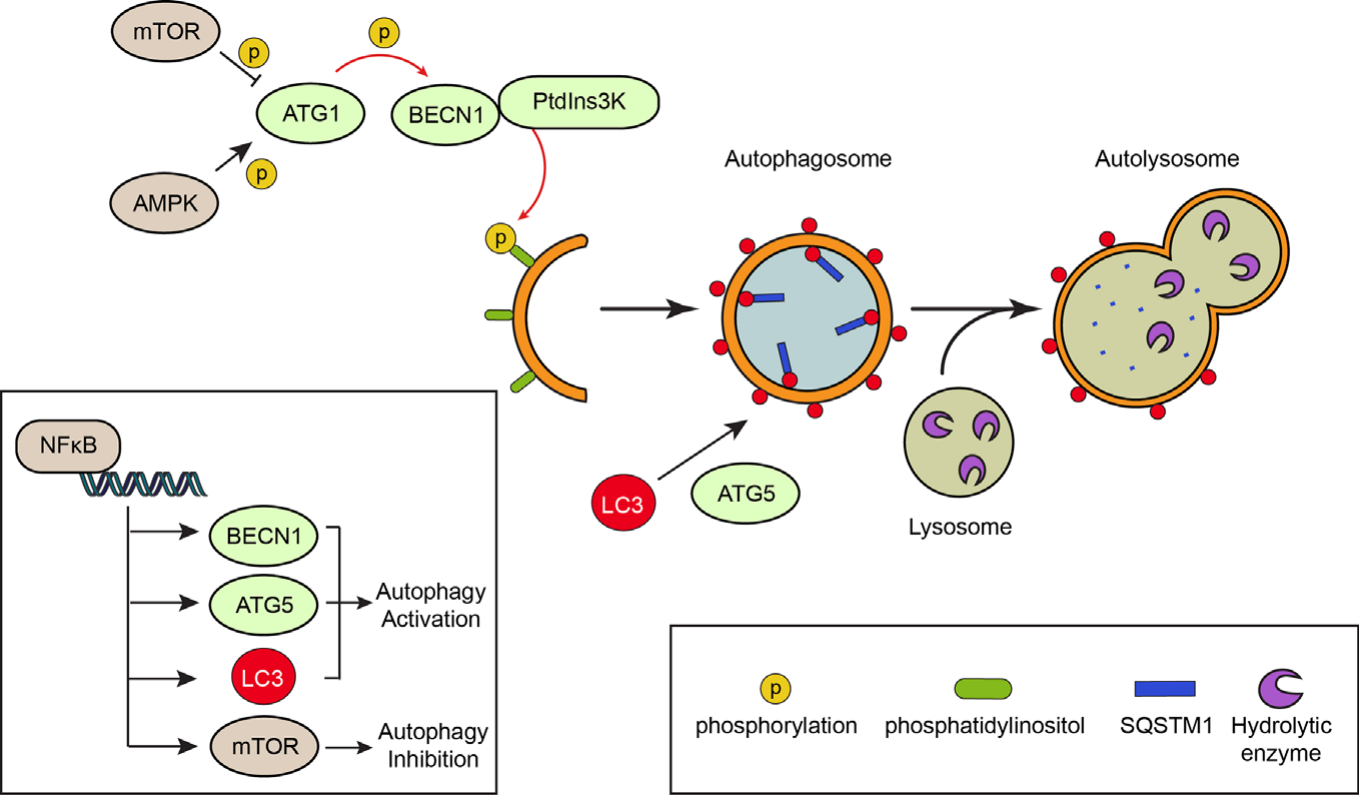
The mechanism of autophagy. Autophagy is a degradation process that involves the formation of double membrane vesicles called autophagosomes. Canonical signaling pathways, such as activation of the nutrient deprivation sensor AMP-activated protein kinase (AMPK) or inhibition of the nutrient-full sensor mechanistic target of rapamycin (mTOR) activate autophagy-related protein 1 (ATG1) kinase by phosphorylation. This in turns phosphorylates the Beclin 1 (BECN1) protein, allowing formation of a PtdIns3K complex that phosphorylates the phosphatidylinositol of intracellular membranes. Formation of the autophagosomes implicates the elongation of the membranes and their decoration with LC3 molecules, a process that is assisted by the ATG5 protein. The material targeted for degradation (i.e. proteins and organelles) arrives to the elongating membrane of the autophagosome through “receptors”, like SQSTM1/p62, which binds polyubiquitinated proteins. Once the autophagosome is formed, it fuses with a lysosome that contains hydrolytic enzymes, leading to the degradation of the material enclosed within the autophagosome. The NFκB pathway is involved in transcriptional up-regulation of autophagy proteins such as BECN1, LC3 and ATG5, thereby promoting autophagy.

Autophagy is controlled by stress signaling pathways that work as an on/off switch. This switch is known as ATG1 (also known as Unc-51-like autophagy activating kinase, ULK1), a kinase activated by AMP-activated protein kinase, AMPK, and inactivated by the mechanistic target of rapamycin, mTOR (64), a serine/threonine protein kinase. Both AMPK and mTOR are kinases that check the nutritional status of the cell. Under normal nutritional conditions, mTOR represses autophagy, while starvation increases AMPK activation and autophagy (65, 66). Thus, starvation and mTOR repression are common autophagy inducers. Active ATG1 phosphorylates and activates Beclin 1 (BECN1), a protein required to transform intracellular membranes into autophagic membranes (67). BECN1 assembles the phosphatidylinositol 3-phosphate kinase complex, PtdIns3K, which catalyzes the formation of phosphatidylinositol 3-phosphate from phosphatidylinositol, serving as an intracellular domain that recruits other ATG proteins that elongate the autophagosome (68). The mechanism of autophagy is depicted in **Figure 3**.

Autophagy may also be controlled by inflammatory pathways like those converging in the transcription factor NFκB (69–71). NFκB is a master regulator of the inflammatory response; the activation of this pathway depends on the degradation of its specific inhibitor, the inhibitor of NFκB (IκB) proteins following their phosphorylation by the IκB kinase IKK complex, allowing NFκB translocation to the nucleus that promotes the expression of genes involved in inflammation, cell proliferation and survival, epithelial-to-mesenchymal transition and invasion, angiogenesis and metastasis (72). Activation of NFκB induces expression of genes involved in autophagy such as BECN1, ATG5 and LC3, leading to increased autophagy (73, 74). However, NFκB may also reduce autophagy by promoting expression of mTOR pathway components (75). Most of the increased NFκB activity observed in solid malignant tumors is due to increased production of IKK-activating cytokines, including tumor necrosis factor, TNF, and IL1β (76). The role of NFκB in autophagy is also shown in **Figure 3**.

Modulation of autophagy is relevant during the pathophysiological progression of cancer. It has been reported that autophagy has a dual role in cancer. Autophagy inhibition in normal tissues leads to tumor formation, while in established tumors increased autophagy is a mechanism that overcomes a nutrient-deficient environment and promotes tumor growth (4). Spontaneous formation of lung carcinoma and hepatocellular carcinoma is observed in mice with a heterologous deletion of BECN1, suggesting that some components of the autophagic machinery may behave as tumor suppressors (77). However, breast carcinoma cells knocked down for BECN1 show decreased tumor growth *in vivo*, indicating that in developed carcinoma cells, BECN1 behaves as an oncogene (78). Similar results have been observed for other ATG proteins, including ATG5 and ATG7 (79, 80).

This dual role of autophagy in cancer is far more complex when considering that tumor cells and non-tumor cells within the tumor microenvironment exhibit different autophagic status, and that autophagy levels of each may vary in time, explaining tumor initiation and progression. This is the case for OSCC, as it will be discussed in the following section.

## The Role of Autophagy in the OSCC Microenvironment

Autophagic impairment is mostly associated with poorer prognosis in patients with OSCC (81). SQSTM1/p62 is highly accumulated in patients with advanced OSCC, suggesting that autophagosomes do not fuse with the lysosome in tumor cells (82). Accumulation of SQSTM1/p62, as well as LC3 and BECN1 in poorly differentiated OSCC, is correlated with immune infiltration of T cells and TAMs, revealing that autophagic inhibition during advanced stages of the OSCC is relevant to establish a tumor immune niche (83). Autophagy seems to inhibit OSCC cell migration, which may explain the reduced autophagic status during advanced OSCC. Indeed, downregulation of ATG7 in OSCC cells augments tumor cell migration through a mechanism dependent on Toll-like receptor 4, TLR4, a protein highly expressed during poor prognosis OSCC (84). TLR4 may activate the NFκB pathway by inhibiting autophagy in OSCC cells (84). However, other studies suggest that autophagy activation is associated with the progression and poor prognosis in OSCC, because higher levels of BECN1, LC3, ATG5, and ATG16L are found in patients with advanced TNM stages (85–88). These observations, however, may be a result of either increased or decreased autophagy, and functional experiments would be required to clearly identify whether autophagy is activated or inhibited instead.

With low oxygen available and intracellular inhibition of autophagy, the preferred metabolic pathway in OSCC cells is glycolysis. High levels of the glycolytic enzyme phosphofructokinase, PFK, are commonly observed in poorly differentiated OSCCs (89). Novel compounds based on a 4H-1-benzopyran-4-ones structure show differential cytotoxicity on OSCC cells by blocking glycolysis, and on CAFs by suppressing the Krebs cycle (90), further indicating that the tumor microenvironment of OSCC depicts different metabolic requirements. Studies suggest that this metabolic difference occurs as a result of a cellular reprograming of normal fibroblasts into CAFs by OSCC cells (91). During this reprogramming, normal fibroblasts export their mitochondria into OSCC cells through tunneling nanotubes, which in turn produce lactate that fuels the fibroblasts and increases HIF1α-dependent transcription, supporting the transformation of normal fibroblasts into CAFs (91). Despite this mitochondrial transfer mechanism, OSCC relies on anaerobic metabolism to produce lactate. AMPK is inactive in the normal fibroblast. However, AMPK activity increases progressively when normal fibroblasts are transformed in CAFs, suggesting that autophagy may be an important factor in the formation of CAFs by OSCC cells (91). CAFs provoke a reduction in the activation of AMPK in OSCC cells, which explains not only the possibly reduced autophagy, but also the resistance to treatment against metformin, a chemical activator of AMPK that increases AMP levels within the cell and reduces tumor development in pancreatic, colorectal and hepatocellular cancer, among others (92, 93).

The low-to-high autophagy in CAFs is interesting and might explain the controversial role of autophagy in chemokine release from OSCC-related CAFs. Autophagy may be required for chemokine release. For instance, chemical inhibition of heat shock protein 90, HSP90, which is well-known to inhibit autophagy by decreasing NFκB-dependent transcription of autophagic genes like *beclin-1* in other cancer models (94), dramatically reduces the release of the CCL7 chemokine by CAFs, thereby decreasing the invasive rate of OSCC cells (95). As mentioned, the NFκB pathway is severely increased in CAFs as a result of IL1β stimulation (derived from OSCC cells), ultimately leading to the release of chemokines from CAFs (38). This suggests that HSP90-mediated autophagy in CAFs may be required for the release of chemokines that promote migration and invasion of OSCC cells. CCL2 and CXCL1 chemokines are released from skin keratinocytes after UV exposure by an ATG7-dependent mechanism (96). Inhibition of autophagy has been related to increased release of IL1β in macrophages (97), while CAF-related chemokines like CCL2 are known to reduce autophagy in breast cancer (98). There may be a vicious cycle between OSCC cells and CAFs; OSCC cells may release IL1β and promote autophagic-dependent release of chemokines by CAFs, which then inhibit autophagy in OSCC cells, raising the levels of IL1β even more. Further experiments would be required to confirm these possibilities.

Autophagy inhibition in OSCC cells can also be regulated by chemokines released from CAFs. For instance, expression of the FLJ22447 long non-coding RNA, lncRNA, is upregulated in CAFs, which prevents autophagic degradation of IL33 by inhibiting SQSTM1/p62 complex formation with the cargos in OSCC cells (26). Given that IL33 supports tumor growth (99), FLJ22447 is an important factor for OSCC cell proliferation (26), indicating that autophagy inhibition in CAFs is relevant for OSCC tumor growth.

Given that CAFs can both inhibit and activate autophagy in OSCC, a conciliated model is proposed, where immature CAFs, with low autophagy, may promote the release of some molecules such as IL33, while mature CAFs, with high autophagy, may promote the release of other cytokines like CCL2. Most importantly, given that OSCC cells direct CAF maturation, OSCC cells up-regulate their growth by modulating autophagy levels in CAFs. This interplay between CAFs and OSCC cells can provide therapeutic cancer resistance. *In vitro* exposure of OSCC cells to cellular stressors such as cadmium or tri-gas hypoxia leads to increased autophagy through mTOR inactivation (100, 101), suggesting that OSCC cells are still sensitive to autophagy under insults. Indeed, the lower basal autophagy in OSCC cells seems to provide a faster and sustained increase in autophagy after treatment with chemotherapeutic agents, such as cisplatin (102). Thus, an essential aspect to improve the chemotherapeutic toxicity over OSCC cells is to deplete the survival response of the tumor cells by concomitantly using an autophagy inhibitor compound like 3-methyladenine (102). The same results have been observed when OSCC cells are challenged with nutrient starvation conditions and co-treated with autophagy inhibitors (103). Given that CAFs reduce autophagy of OSCC cells, it is fair to conclude that CAFs promote the phenotype change of OSCC cells toward treatment-resistant cells. The phenotypic change of OSCC cells is associated with the expression of the membrane marker CD24, which correlates with both chemotherapy resistance and autophagy sensitivity (104). Curiously, while chemotherapy increases autophagy in OSCC cells, it decreases autophagy in CAFs, as reflected by higher accumulation of SQSTM1/p62 levels in α-SMA-containing cells (105).

To date, the role of autophagy in OSCC-related TAMs remains unclear. However, two recent studies have shown that OSCC cells can secrete high levels of the receptor activator of nuclear factor κB ligand, RANKL, a protein that transforms macrophage-derived pre-osteoclasts into osteoclasts, therefore promoting bone resorption and metastasis (106, 107). Interestingly, RANKL also binds to its receptor on OSCC cells, leading to a slight increase in autophagy that provides resistance against extrinsic apoptotic pathway inducers like the tumor necrosis factor-related apoptosis-inducing ligand, TRAIL (108). Caspase activation is observed when OSCC cells are stimulated with TRAIL in the absence of RANKL (108). This supports the view that reduced basal autophagy in OSCC cells is required to respond promptly against a stressful condition and to cope with a death stimulus.

**Figure 4:**
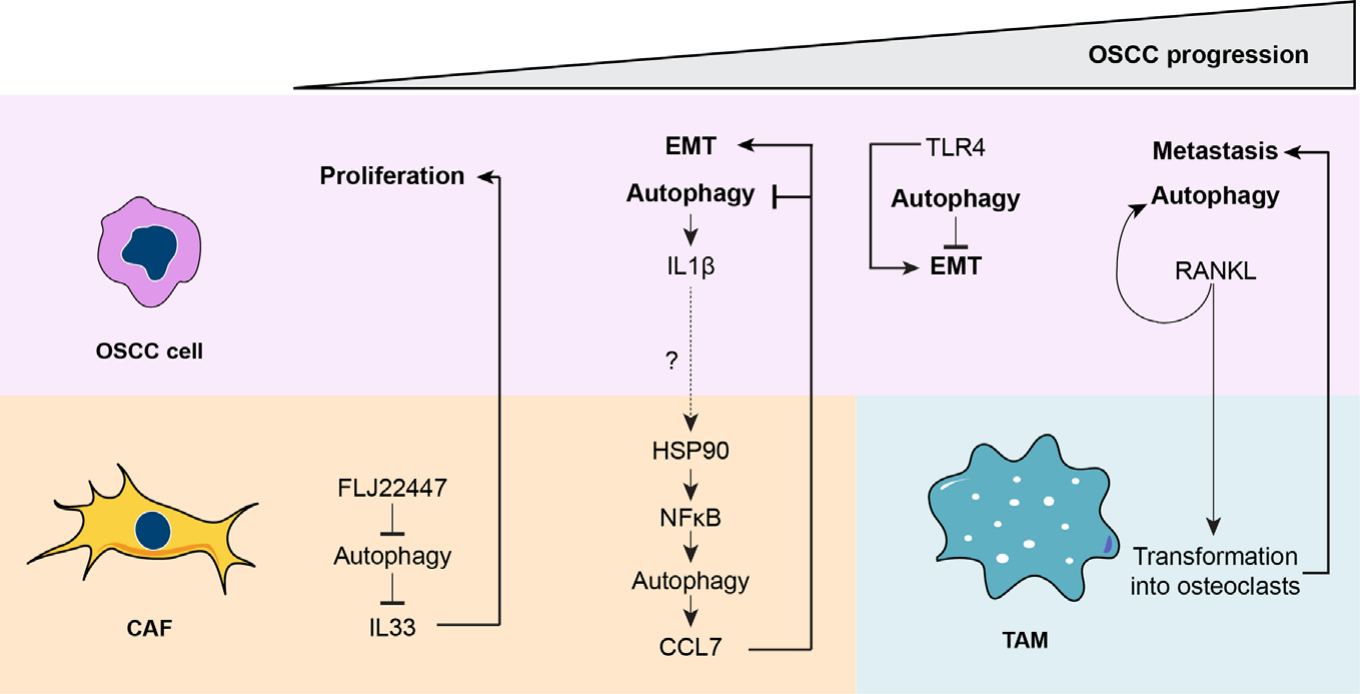
A unified model for the role of autophagy during oral squamous cell carcinoma (OSCC) progression. During the early stages of OSCC, the long non-coding RNA FLJ22447 inhibits autophagy in cancer-associated fibroblasts (CAFs), impairing the autophagic degradation of IL33. Increased levels of IL33 are released from CAFs, inducing proliferation of the OSCC cells. Then, OSCC cells increase autophagy that releases interleukin-1β (IL1β), which then increases autophagy in CAFs through an nuclear factor κB ((NFκB)-dependent mechanism. The IL1β released from the OSCC cells may promote activation of heat shock protein 90 (HSP90) in the CAFs to increase NFκB activity, but this needs to be demonstrated (dashed line). However, it is known that NFκB-dependent autophagy in CAFs induces release of chemokine (C-C motif) ligand 7 (CCL7), which acts on OSCC cells, promoting epithelial-mesenchymal transition (EMT) and inhibiting autophagy. During later phases of OSCC, autophagy reduces Toll-like receptor 4 (TLR4)-dependent EMT. Therefore, inhibition of autophagy by CCL7 may promote TLR4-depedent EMT. Finally, during the advanced stages of OSCC, receptor activator of nuclear factor κB ligand (RANKL), which induces autophagy in OSCC cells, is released from OSCC cells transforming tumor-associated macrophages, TAMs, into osteoclasts, ultimately inducing metastasis.

Together, autophagy is a key process to induce CAF maturation. The different levels of autophagy in CAFs provide a delicate tuning process that regulates the cytokines released in a time-specific manner. The maturation of CAFs is directed by OSCC cells; thereby OSCC cells control their malignancy by indirectly promoting the differential chemokine release from CAFs. Importantly, the lower the autophagic status of OSCC cells, the more malignant the tumor is and more resistant to chemotherapy. Increased sensitivity to cell death is achieved by co-treatment with chemotherapy and autophagy inhibitors, suggesting that malignant OSCC also respond better to autophagy-based treatments. Finally, OSCC cells release molecules that increase their autophagy status through an autocrine mechanism and modulate TAMs that promote metastasis. The role of autophagy during OSCC progression is depicted in **Figure 4**.

## Concluding Remarks

The number of patients with OSCC keeps rising, because the risk factors involved in the development of OSCC are mainly environmental and considered to be “normal” by the modern society, such as tobacco smoking and alcohol consumption. Although OSCC is observed as morphological changes in oral epithelial cells, other cells in the oral tissue are also exposed to these risk factors. Efforts have been made during the last decade to elucidate the role of CAFs and immune cells in OSCC. These cells communicate with the OSCC cells and receive instructions from them by a complex cytokine interplay. Tobacco, alcohol and infection with HPV behave as direct tissue stressors modulating the homeostatic cellular process known as autophagy. Autophagic degradation levels are cell-specific and time-specific; OSCC cells show a counterintuitive reduction of autophagy over time, while CAFs show progressive increase in autophagy over time. This autophagic balance is important to induce the structural change from normal fibroblasts into CAFs that control the release of cytokines from CAFs and promote EMT in OSCC cells. Thus, autophagy is a critical player in the crosstalk between OSCC cells and tumor microenvironment cells such as CAFs and TAMs, fine-tuning the development of the OSCC.

## Author Contributions

Original draft writing by DP-O, MR, MH-C, CK, EM, SL, VT, and AC. Review and editing by DP-O, CAR-S, SL, VT, and AC. All authors contributed to the article and approved the submitted version.

## Funding

This work was supported by the Agencia Nacional de Investigación y Desarrollo (ANID, Chile): FONDECYT (3200313 to DP-O) (1200499 to EM) (1201684 to CAR-S) (1180495 to VT) (1171075 to AC); PIA-ANID (ACT172066 to EM and AC); FONDAP (15130011 to SL, VT, and AC); ANID PhD fellowship (21140848 to CK). Also supported by the U-Inicia Program of the Universidad de Chile (UI-024/19 to MR).

## Conflict of Interest

The authors declare that the research was conducted in the absence of any commercial or financial relationships that could be construed as a potential conflict of interest.

## Abbreviations

α-SMA, alpha smooth muscle actin; AMPK, AMP-activated protein kinase; ATG, autophagy-related protein; BECN1, Beclin 1; CAF, cancer-associated fibroblast; CCL, chemokine (C-C motif) ligand; CCR, chemokine (C-C motif) receptors; CXCL, chemokine (C-X-C motif) ligand; CD, cluster of differentiation; DLC-1, Deleted in Liver Cancer; EGF, epithelial growth factor; EMT, epithelial-mesenchymal transition; FAK, focal adhesion kinase; HSP90, heat shock protein 90; HPV, human papillomavirus; HIF1α, hypoxia inducible factor 1 subunit alpha; IκB, inhibitor of NFκB; IL, interleukin; IL1β, interleukin-1β; JAK2, Janus kinase 2; lncRNA, long non-coding RNA; mTOR, mechanistic target of rapamycin; MMP, metalloproteinase; miR, microRNA; MAP1LC3/LC3, Microtubule Associated Protein 1 Light Chain 3; MCP-1, monocyte chemoattractant protein-1; MDX1, Myc-competitor MAD; NFκB, nuclear factor κB; OSCC, oral squamous cell carcinoma; PtdIns3K, Phosphatidylinositol 3-phosphate kinase complex; PFK, phosphofructokinase; PDGF-Rβ, platelet-derived growth factor receptor β; RANKL, receptor activator of nuclear factor κB ligand; ROCK2, Rho-associated coiled-coil kinase 2; SQSTM1/p62, Sequestrosome 1; STAT3, signal transducer and activator of transcription protein 3; TLR4, Toll-like receptor 4; TGFβ, transforming growth factor β; TAM, tumor-associated macrophage; TAN, tumor-associated neutrophil; TNF, tumor necrosis factor; TRAIL, tumor necrosis factor-related apoptosis-inducing ligand; ULK1, Unc-51-like autophagy activating kinase; VEGF, vascular endothelial growth factor.
